# Bilateral vestibulopathy in Alexander disease type II– a case report

**DOI:** 10.1007/s00405-025-09416-7

**Published:** 2025-05-06

**Authors:** Jan Bernhard Hofmann, Matthias Gautschi, Anja Vossenkaul, Marisa Blanquet, Tatiana Bremova-Ertl

**Affiliations:** 1 Department of Neurology, University Hospital Bern (Inselspital) and University of Bern, Bern, 3010 Switzerland; 2 Division of Pediatric Endocrinology, Diabetes and Metabolism, Department of Paediatrics, Inselspital, Bern University Hospital, University of Bern, Bern, 3010 Switzerland; 3 Institute of Clinical Chemistry, Inselspital, Bern University Hospital, University of Bern, Bern, 3010 Switzerland; 4 Division of Neuropediatrics, Development and Rehabilitation, Department of Pediatrics, Inselspital, Bern University Hospital, University of Bern, Bern, 3010 Switzerland; 5 Center for Rare Diseases, University Hospital Bern (Inselspital) and University of Bern, Bern, 3010 Switzerland

**Keywords:** Alexander disease, Bilateral vestibulopathy, Brainstem atrophy, Vestibulo-ocular reflex, 4-aminopyridine

## Abstract

**Introduction:**

Alexander disease (AxD) is rare leukodystrophy caused by a mutation in the glial fibrillary acidic protein (GFAP) gene. Astrocyte dysfunction leads to myelinization disturbances and white matter damage, resulting in distinct neurological symptoms and neuroradiological findings.

**Case report:**

Our patient, a 46-year old male, showed typical symptoms of AxD, including myoclonus of the soft palate, nystagmus, and cerebellar ataxia, as well as typical radiological findings found in AxD. The results of a device-based vestibular examination, including a video head-impulse test, showed a bilaterally decreased gain of vestibulo-ocular reflex of all semicircular canals suggestive of a bilateral vestibulopathy (BVP), a novel aspect of AxD. Symptomatic treatment of cerebellar ataxia and BVP with 4-aminopyridine (4-AP) led to an improvement of several device-examined vestibular parameters, but without subjective improvements in balance.

**Conclusion:**

This case report describes BVP in a patient suffering from AxD, a novel phenotype of the disease. In leukodystrophies, such as AxD, central vestibular symptoms should be assessed early on to evaluate the potential use of 4-AP.

**Supplementary Information:**

The online version contains supplementary material available at 10.1007/s00405-025-09416-7.

## Introduction

Alexander disease (AxD) is an ultra-rare form of leukodystrophy caused by a dominant mutation in the glial fibrillary acidic protein (GFAP) gene [1]. This mutation leads to the pathological accumulation of GFAP in astrocytes, called Rosenthal fibers [1]. By impairing the function of diverse cell types in the CNS [2], astrocyte dysfunction causes disturbances in myelinization and damages in the white matter [1].

There are two types of AxD, type I and type II, characterized by the age of onset, symptoms, and MRI findings [3]. Type I has an early onset (in many cases before the age of 4 years) and typically presents with seizures, macrocephaly, encephalopathy, paroxysmal deterioration, or developmental delays [3]. Type II can manifest at any age; common findings include autonomic dysfunction, oculomotor and bulbar symptoms, or palatal myoclonus [3]. Common MRI findings in AxD include white matter changes with frontal predominance, a periventricular rim with a high T1 signal and low T2 signal, abnormalities of basal ganglia, thalami or brain stem, as well as contrast enhancement of particular gray and white matter structures [4].

The diagnosis of AxD involves a suspected diagnosis based on neurological and neuroradiological findings, confirmed by genetic analysis with regards to the GFAP mutation. To date, there are no disease-modifying treatments available. The median age of survival is 14 years for type I AxD and 25 years for type II AxD from the date of diagnosis [3].

This case report aims to describe the bilateral vestibulopathy (BVP) as a novel aspect of the AxD phenotype, as well as an individual therapeutic approach for disease-related central vestibular symptoms, using the potassium-channel blocker 4-aminopyridine (4-AP), in a patient suffering from AxD type II. This case report is intended to encourage the description of further aspects of AxD, which is essential to enable an early diagnosis and therapy in rare diseases.

### Case report

**Fig. 1:**
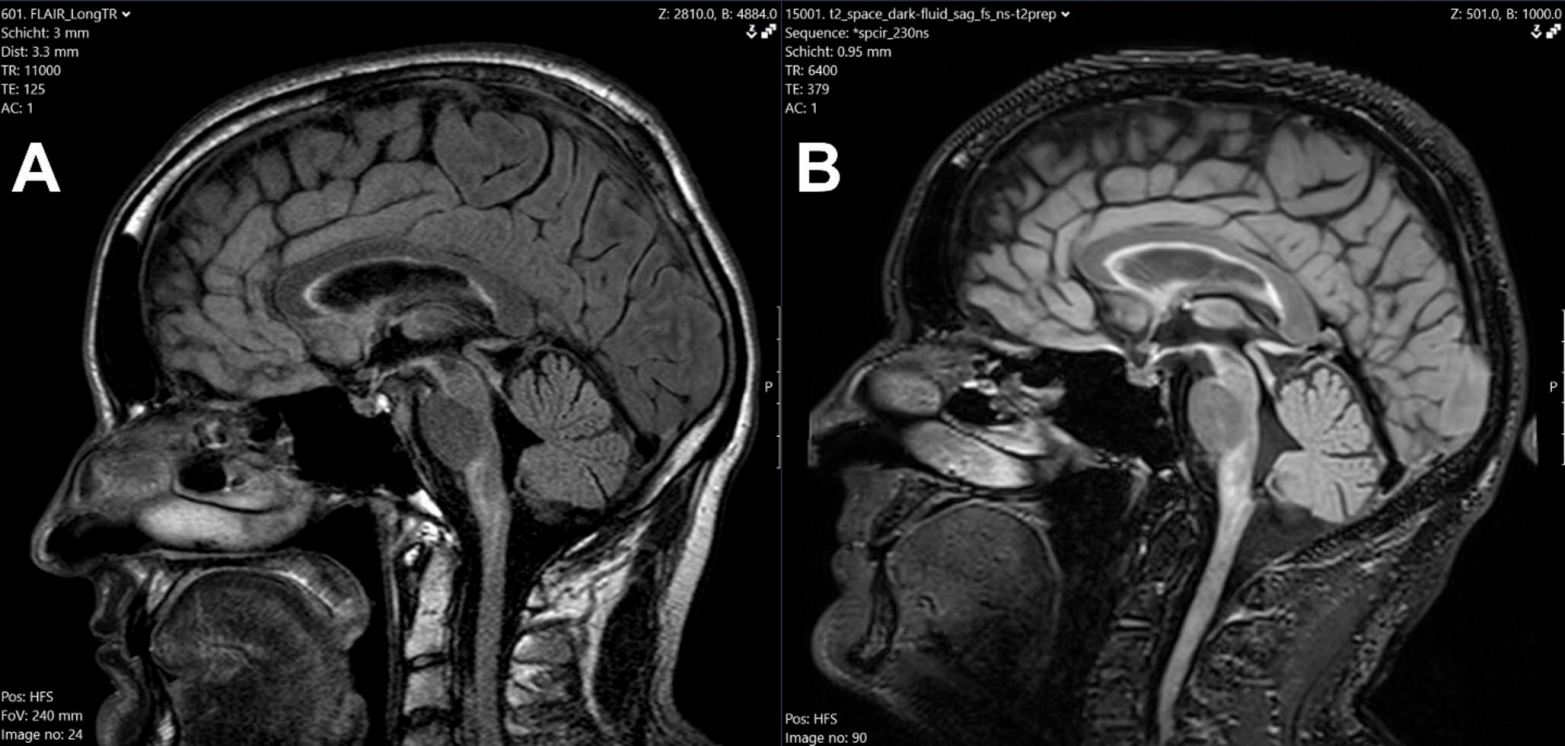
MRI findings (FLAIR) at the age of 32 (**A**) compared to the findings at the age of 45 (**B**)

At the age of 32, a man developed a self-described “eye wobble”, interpreted as pendular nystagmus and impaired balance. An MRI (Fig. 1A) showed discrete atrophy of the medulla oblongata with patchy T2- and FLAIR hyperintensities. In addition, hyperintensities in the basal ganglia, cerebral peduncles, cerebellum, pons, and spinal cord were found. At the time, a diagnosis could not be assigned based on the clinical findings or MRI results. As the nystagmus resolved, no further assessments were performed, even though a subtle balance impairment remained.

Thirteen years later, at the age of 45, the patient presented with additional symptoms that had developed over the past years. These included a twitching Adam’s apple (Video 1), problems with speech and articulation, and worsened imbalance but without falls. He noticed urge incontinence with rare nocturia and sleep disturbances—including snoring and daytime sleepiness, paresthesia (“prickling”) in the limbs, and twitching in the upper arm.

Upon neurological examination, we found palatal myoclonus, also called myorhythmia with a frequency of one per second, dysarthrophonia, a weak gag reflex, weak reflexes of the lower extremities, and discrete truncal ataxia.

The MRI (Fig. 1B) showed a slightly progressive atrophy of the medulla oblongata and a subtle volume reduction of the cervical cord with a preservation of the pontine volume, the so-called tadpole sign [5]. These findings, combined with the neurologic symptoms, were highly suspicious of a diagnosis of Alexander disease (AxD) type II. A mutation analysis of the GFAP gene revealed the known [6] pathogenic variant c.208 C > T p.(Arg70Trp) rs60343255, confirming the diagnosis.

A device-based examination battery was performed. A video head-impulse test (vHIT) showed bilaterally decreased vestibulo-ocular reflex (VOR) of all semicircular canals (SCC). Besides a slight downbeat nystagmus, the video-oculography was normal. The patient showed reduced gait velocity and increased gait variability in automated gait analysis (GAITRite^®^), without complaining of oscillopsia. A posturography showed impaired postural stability, emphasized on soft ground. The caloric irrigation testing was normal. We interpreted these findings as a BVP due to the involvement of the vestibular nuclear area in the context of the brainstem degeneration, however peripheral or ganglionic (Scarpa`s ganglion) causes could not be excluded. This is a novel aspect of the AxD phenotype.

Based on a combination of axial cerebellar ataxia with BVP, a focused physical therapy program together with 4-aminopyridine (4-AP), a blocker of Kv1 voltage-activated potassium-channels [7], was introduced [8]. 4-AP increases the excitability of neurons, particularly of cerebellar Purkinje cells and other cerebellar cells [8–10]. Aminopyridines can be used for the symptomatic treatment of certain forms of cerebellar ataxia [11].

**Fig. 2:**
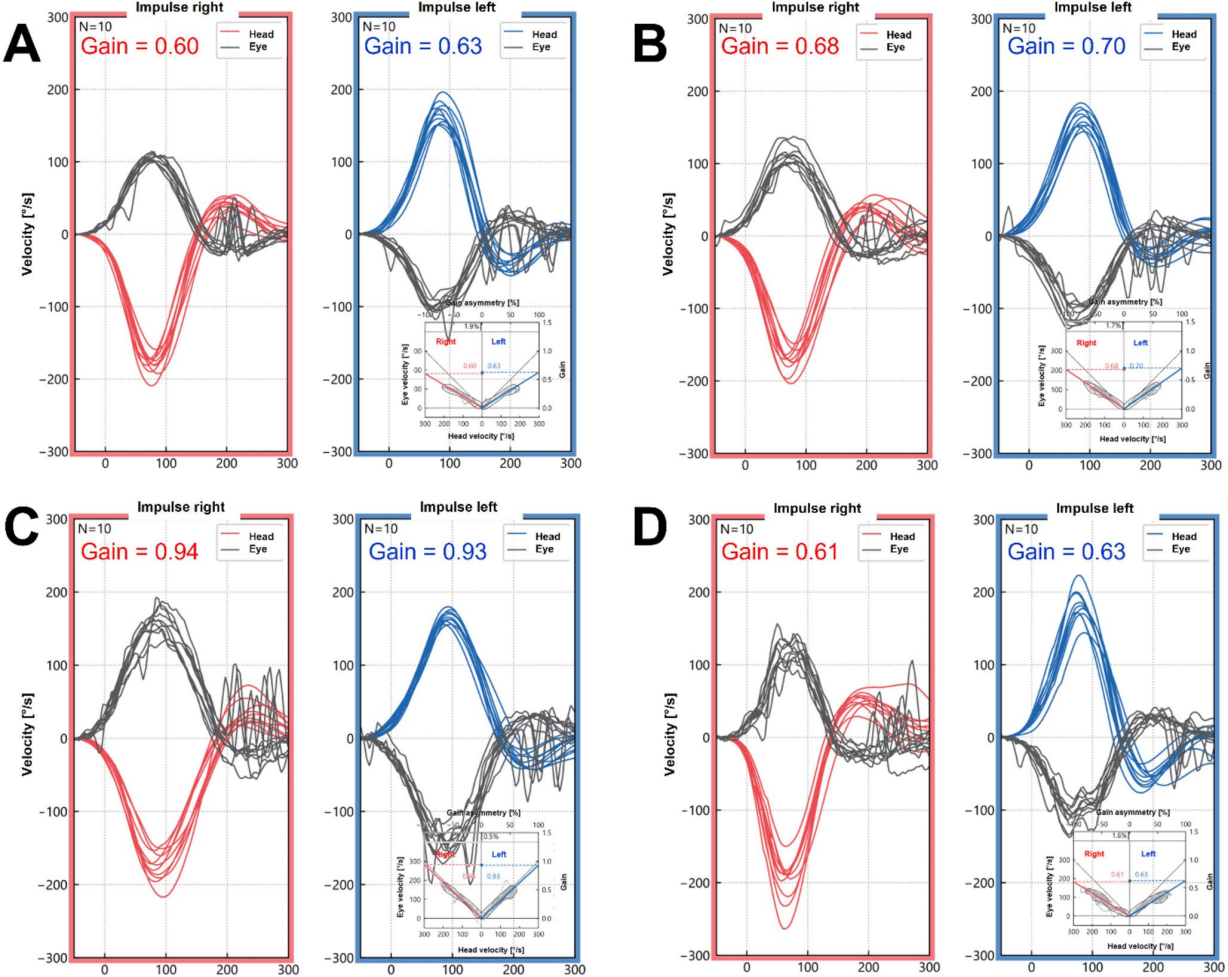
vHIT gains of the horizontal canals with and without 4-AP measured at different timepoints: once before (**A**) and after (**B**) the intake of 4-AP on the same day, 4 weeks after the start of 4-AP therapy (**C**), and one year after the first vHIT examination (**D**)

Interestingly, this led to an improvement of the VOR: We performed two vHITs on the same day, once before (Fig. 2A) and once after (Fig. 2B) taking 4-AP, as this is a short-acting symptomatic agent. In all SCC, the vHIT gain increased after taking the medication. GAITRite^®^ showed a slight improvement in gait variability with an unchanged impairment of gait under sensory-challenging conditions (e.g., closed eyes). Posturography showed a minimally improved postural stability. After 4 weeks of treatment, the vHIT gain increased in all SCC (Fig. 2C). However, the patient reported no subjective improvement in balance, so the treatment was stopped. No adverse events were described.

One year later, at the age of 46, the vHIT gain reached the original level in the horizontal canals (Fig. 2D) and worsened in the anterior and posterior canals, compared to the first examination. The patient reported a worsening of balance over the year.

The patient’s condition currently remains stable with regular physical activity and targeted balance and coordination exercises (Table 1).

**Table 1:** Timeline

Age (years)	Symptoms and clinical findings	Assessments	Treatments
**32** No diagnosis made	**Initial symptoms** • “eye wobbling”; resolved over time • Mild balance impairment	**MRI** • Atrophy and T2 hyperintensities in the medulla oblongata • T2 hyperintensities in basal ganglia, cerebral peduncles, cerebellum, pons, spinal cord	
**32–45**	**Successive symptom onset** • Twitching of the Adam’s apple • Articulation problems • Increased imbalance (without falls) • Urge incontinence, nocturia • Sleep disturbances (snoring, daytime sleepiness, limb paraesthesia, arm twitching)		
**45** Diagnosis of AxD	**Findings** • Palatal myorhythmia (1 Hz) • Dysarthrophonia • Weak gag reflex • Weak reflexes of the lower extremities • Discrete truncal ataxia	**MRI** • Progressive atrophy of the medulla oblongata and subtle volume reduction of the cervical cord with a preservation of the pontine volume (“tadpole sign” [5]) **GFAP gene mutation analysis**: **diagnosis of AxD** • c.208 C > T p.(Arg70Trp) rs60343255	
Before and 4 h after 4-AP intake		**Vestibular examination battery** ***before*** **4-AP** • vHIT: bilaterally decreased VOR gain in all SCC • VOG: normal (beside slight downbeat nystagmus) • GaitRite^®^: reduced gait velocity, increased gait variability, empathized on uneven ground (without complaining oscillopsia) • Posturography: impaired postural stability • Caloric irrigation: normal **Vestibular examination battery** ***4 h after*** **4-AP** • vHIT: gain increase in all 6 SCC • GaitRite^®^: slightly improved gait variability • Posturography: minimally improved postural stability	**Treatment facing cerebellar ataxia and BVP** • 4-Aminopyridine (4-AP) • Focused physical therapy
4 weeks on 4-AP	• No subjective improvement of imbalance	**Vestibular examination battery** ***4 weeks*** **on 4-AP therapy** • vHIT: gain increase in all 6 SCC	• Stop of 4-AP treatment due to a lack of subjective balance improvement. No described adverse events. • Focused physical therapy
**46**	• Increasing imbalance over the last year	**Vestibular examination battery** ***1 year after diagnosis*** • vHIT: decrease to original gain level in horizontal SCC, worsening in the anterior and posterior SCC compared to the first examination	• Focused physical therapy

## Discussion

Our patient shows classical symptoms of AxD type II, including a myoclonus of the soft palate, dysarthorphonia, cerebellar ataxia, and autonomic involvement.

The BVP described in this case report is a novel aspect of the AxD phenotype. The causes of BVP are usually labyrinth damage, e.g., chemotoxic by antibiotics, or inflammatory, either infectious (meningitis) or autoimmunological (e.g., Cogan syndrome). Central causes such as neurometabolic conditions (e.g., Gaucher disease) [12] or Wernicke encephalopathy [13] are rare. The profound brainstem atrophy is suggestive of a central cause of BVP. The highest levels of GFAP-positive astrocytes are found in the medulla oblongata, cervical spinal cord and hippocampus [14]. Therefore, the pathological accumulation of Rosenthal fibres [1] in the context of AxD, leading to astrocyte dysfunction, is a further possible explanation for a central cause of the VOR impairment.

4-AP treatment led to an increase of the vHIT gain after the first dose and a subsequent further increase after one month of therapy. The effect of 4-AP on VOR gain has been previously described [15, 16]. 4-AP blocks Kv1.1 voltage-dependent potassium channels, which play a key role in encoding transient motion components in VOR circuit elements [17]. However, the therapy did not improve the patient’s subjective imbalance, leading to the attenuation of the therapy.

As AxD is an ultra-rare form of leukodystrophy, there is a need to illustrate uncommon observations of its phenotype as well as potentially effective symptomatic therapeutical interventions, even in individual cases. To our knowledge, neither a BVP nor a trial of a 4-AP treatment on central vestibular symptoms in patients suffering from AxD have been previously described.

## Conclusion

We described BVP as a novel phenotype of AxD as well as a potential therapeutic approach with 4-AP for associated central vestibular symptoms. This case report shows that central vestibular involvement can contribute to motor impairment and imbalance in leukodystrophies, such as AxD. Therefore, central vestibular symptoms, in particular cerebellar ataxia and BVP, should be assessed early on in leukodystrophies to evaluate the potential use of 4-AP.

## Electronic supplementary material

Supplementary Material 2: Video 1: Twitching of the Adam’s apple.
